# COVID-19 Pneumonia Presenting As Solitary Pulmonary Lesion

**DOI:** 10.7759/cureus.47876

**Published:** 2023-10-28

**Authors:** Herik Valles-Bastidas, Alejandro J Torre-De León, Horiana B Grosu

**Affiliations:** 1 School of Medicine and Health Sciences, Tecnologico de Monterrey, Monterrey, MEX; 2 Department of Pulmonary Medicine, MD Anderson Cancer Center, Houston, USA

**Keywords:** lung mass, ct imaging, lung cancer, pneumonia, covid-19

## Abstract

Common findings of COVID-19 pneumonia on chest CT images include ground-glass opacities and organizing pneumonia. Here, we present a patient with a history of lung cancer who came to our center for re-staging CT studies, which showed a solitary peripheral lung mass suggestive of lung cancer. While being evaluated for the mass, the patient developed respiratory failure due to COVID-19 pneumonia. After treatment for COVID-19 and recovery, CT showed complete resolution of the solitary peripheral lung mass. This case highlights that COVID-19 can, on occasion, present with CT findings that mimic those of lung cancer.

## Introduction

Symptoms of COVID-19 typically include cough, fever, anosmia, dysgeusia, nausea, diarrhea, and dermatologic findings. In the most severe cases, patients develop pneumonia, acute respiratory distress syndrome, and respiratory failure. Risk factors for severe COVID-19-associated pneumonia and respiratory failure include lack of vaccination, advanced age, and medical comorbidities [1]. In patients with COVID-19 pneumonia, CT imaging of the chest often shows features of organizing pneumonia, such as ground-glass opacities with or without mixed consolidation, mainly distributed throughout the lower lobes [2].

However, a substantial proportion of SARS-CoV-2 infections are detected in individuals who have no symptoms of COVID-19 [3]. Here, we describe a patient with no COVID-19 symptoms and a history of lung adenocarcinoma who on re-staging chest CT scans was found to have a solitary peripheral lung mass suspicious of cancer. During his evaluation for this mass, he developed respiratory failure due to severe COVID-19 pneumonia.

## Case presentation

**Figure 1:**
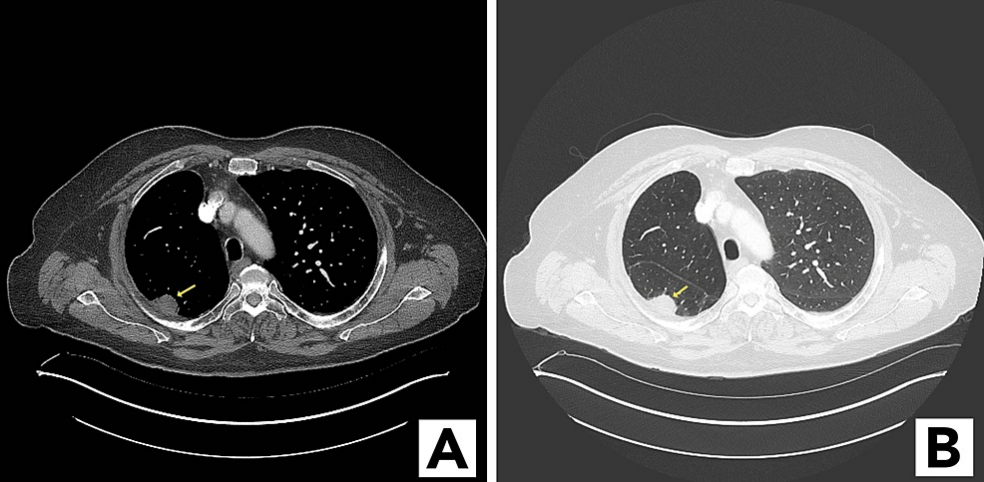
CT images showing nodular subpleural mass (arrow) in the right lower lobe. (A) Mediastinal window; (B) lung window

A 75-year-old male with no respiratory symptoms was referred to the pulmonology department at our institution for a biopsy of a lung mass found on a CT scan performed for re-staging of a previously treated right upper lobe adenocarcinoma. The COVID-19 vaccines were not yet available. He had a history of chronic obstructive pulmonary disease and had undergone a right upper lobectomy for curative treatment of the adenocarcinoma two years earlier. His previous re-staging CT scan, which was performed six months earlier, showed no abnormalities, but this new CT showed a new nodular subpleural mass in the right lower lobe (Figure 1A-1B). In view of this finding and the patient’s history, he was referred to us for consideration for a biopsy.

Before the biopsy, due to the ongoing COVID-19 pandemic, testing for SARS-CoV-2 was ordered. The SARS-CoV-2 RT-PCR test showed a positive result, and further evaluation with bronchoscopic biopsy for the lung mass was postponed until the two-week quarantine time was completed. Three days later, the patient was hospitalized with shortness of breath. He developed severe COVID-19 pneumonia that resulted in hypoxemic respiratory failure and the need for noninvasive positive pressure ventilation.

**Figure 2:**
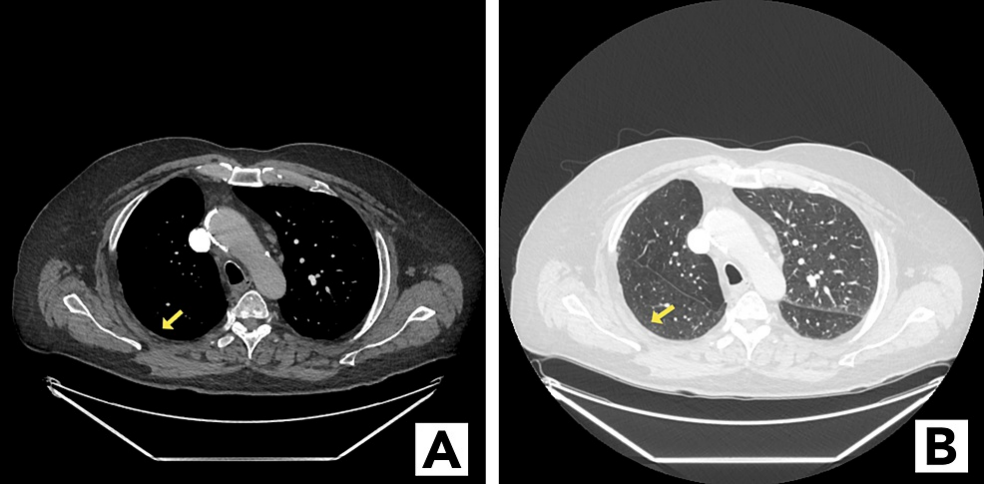
CT images six months later showing complete resolution of nodular subpleural mass in the right lower lobe (arrow). (A) Mediastinal window; (B) lung window

One month after hospitalization, a CT scan showed a significant decrease in the size of the pleural base mass; six months later, CT showed complete resolution (Figure 2A-2B). The mass-appearing lesion was consistent with organizing pneumonia due to COVID-19.

## Discussion

CT findings for COVID-19 do not usually resemble a lung mass. If a patient with no respiratory symptoms and a history of lung cancer presents with a new mass-like lesion on the chest CT, neoplasia or metastasis, rather than infection, is usually the first diagnostic impression. Moreover, previous reports have described the difficulty distinguishing COVID-19 pneumonia from other infectious and noninfectious pneumonia, which may have similar clinical and imaging characteristics. In particular, ground-glass opacities are seen in a wide variety of diseases, mainly in interstitial pneumonia [4]. In general, when we treat patients with rapidly progressive hypoxia, bilateral interstitial infiltrates, and response to steroids, we think of idiopathic interstitial lung diseases. When the radiologic findings show a peripheral predominance of infiltrates and eosinophilia, we narrow this to organizing pneumonia or eosinophilic pneumonia. One deciding factor for organizing pneumonia is the localization of infiltrates in the bases and subpleural regions along with mass-like lesions, like our case [5]. In general, when we treat patients with rapidly progressive hypoxia, bilateral interstitial infiltrates, and response to steroids, we think of idiopathic interstitial lung diseases. When the radiologic findings show a peripheral predominance of infiltrates and eosinophilia, we narrow this to organizing pneumonia or eosinophilic pneumonia. One deciding factor for organizing pneumonia is the localization of infiltrates in the bases and subpleural regions along with mass-like lesions, like our case [5]. A meta-analysis including 15 descriptive studies with 2,451 patients concluded that the most common CT findings for COVID-19 pneumonia are ground-glass opacities, vascular enlargement, interlobular septal thickening, and subpleural bands [6]. Involvement of multiple lobes, particularly the lower lobes, is also reported in most patients with COVID-19 pneumonia [7]. Thus, our patient’s main imaging findings were rare and atypical of COVID-19 infection.

## Conclusions

Mass-like lesions found on CT imaging can be suggestive of COVID-19-related organizing pneumonia. COVID-19 should be included as a differential diagnosis when assessing patients with these findings, especially when symptoms are present.
